# Are emojis ready to promote the WHO 5 moments for hand hygiene in healthcare?

**DOI:** 10.1186/s13756-022-01164-1

**Published:** 2022-10-26

**Authors:** Nasim Lotfinejad, Ermira Tartari, Julien Sauser, Carolina Fankhauser-Rodriguez, Daniela Pires, Didier Pittet

**Affiliations:** 1 Infection Control Programme, Faculty of Medicine, University of Geneva Hospitals, Gabrielle-Perret-Gentil 4, 1205 Geneva, Switzerland; 2 Infection Control Programme, WHO Collaborating Centre on Infection Prevention and Control & Antimicrobial Resistance, Faculty of Medicine, Geneva University Hospitals, Geneva, Switzerland; 3 Faculty of Health Sciences, University of Malta, Msida, Malta

**Keywords:** Emoji, Hand hygiene, Infection prevention and control, Questionnaire, WHO 5 moments

## Abstract

**Background:**

Hand hygiene is universally recognized as a cornerstone measure for the prevention of healthcare-associated infections. Although the WHO “My five Moments for hand hygiene” poster has been used for more than a decade to delineate hand hygiene indications and promote action, adherence levels among healthcare workers are still notoriously low and disquieting. To compensate for the lack of effective hand hygiene communication, we aimed to evaluate emojis as possible surrogates for the non-verbal aspects of hand hygiene behaviour.

**Methods:**

Following a thorough review of the Unicode version 12.0, the most applicable emojis to the terms used in the WHO 5 Moments poster were extracted. We developed a self-administered questionnaire to assess the view of infection prevention and control (IPC) practitioners regarding the use of emojis to show the WHO 5 Moments. Completed questionnaires were collected and analysed to determine the suitability of the existing emojis to illustrate a unified emoji poster. Data were analysed using R (version 3.6.3).

**Results:**

A total of 95 IPC practitioners completed the questionnaire from May to October 2019 from different countries. Of these, 69 (74%) were female, and the mean age of the participants was 44.6 ± 10.87 years. We found appropriate emojis for six of the words used in the poster, including  for touching (72%),  for patient (63%),  for clean (53%),  for procedure (56%),  for body fluid (58%), and  for exposure risk (71%). The existing emojis proposed for the words “hygiene”, “aseptic”, and “surrounding” seemed to be less satisfactory.

**Conclusions:**

In summary, the findings of this study indicate that the existing emojis may not be able to substitute the words used in the WHO 5 Moments poster. Emojis might be helpful to address hand hygiene indications in healthcare that may eventually play a role in promoting this measure. However, emojis should be further studied to choose the most appropriate ones and avoid ambiguity and misinterpretation. More emojis to convey health related messages are needed. We recommend further research in this area to evaluate the effect of using emojis in healthcare-related behaviours.

**Supplementary Information:**

The online version contains supplementary material available at 10.1186/s13756-022-01164-1.

## Background

Healthcare-associated infections (HAIs) are globally recognized among the most common adverse events in care delivery with significant mortality and financial burden for health systems [1]. The World Health Organization (WHO) has estimated that 4.5% of hospitalized patients acquire HAIs in the USA, and 7.1% in Europe [2]. However, the situation is more worrisome in low-resource settings, with approximately 15.5% of patients acquiring a HAI, although the rates may be even higher, mainly due to underreporting [1]. Hand hygiene is known as the global standard of care and one of the most effective measures of infection prevention and control (IPC) [3–5]. During the COVID-19 pandemic, hand hygiene with alcohol-based hand rub was considered globally as one of the most effective, simple and low-cost procedures against COVID-19 cross-transmission [6]. Despite being a simple procedure, hand hygiene adherence levels remain insufficient, and improving compliance has been challenging notwithstanding the multitude of interventions [7, 8]. Hand hygiene compliance levels of 40% have been reported from high-income countries, while this rate is even lower in low- and middle-income countries [1, 9]. WHO developed the Multimodal Hand Hygiene Improvement Strategy to translate hand hygiene guidelines into practice at the point of care. The multimodal strategy includes five key elements: system change, education, evaluation and feedback, reminders in the workplace, and institutional safety climate [10].

Using hand hygiene reminders at the point of care is essential to constantly remind healthcare workers (HCWs) to perform hand hygiene at appropriate indications. Considering the variety of reminders in hospitals, a reminder is deemed to be effective when it stands out from the visual information overload [11]. The WHO “My five moments for hand hygiene” poster is used worldwide as a reminder for HCWs on the indications when hand hygiene should be performed in a healthcare setting [12]. This poster includes five critical moments for hand hygiene action organised in a sequence throughout the delivery of care: Moment 1: before touching a patient; Moment 2: before clean/aseptic procedures; Moment 3: after body fluid exposure/risk; Moment 4: after touching a patient; and Moment 5: after touching patient surroundings. Although visual reminders are helpful to improve hand hygiene, the most effective design to reinforce behavioural change has not yet been identified [13].

One of the significant barriers to effective communication is the fact that text-based communication could be interpreted in many ways for different people. At the same time, individuals who talk face-to-face have the use of intonations, gestures and facial expressions to put their words into context. Emojis, defined as picture characters, could be a solution to add that emotional context to the written communication [14, 15]. A recent study indicated that the use of happy and sad emojis effectively improves hand hygiene behavior, since positive behavior was reinforced by the appearance of happy emojis on alcohol-based hand rub dispenser every time hand hygiene was performed [16]. We hypothesized that emojis might improve hand hygiene compliance when replaced with the words used in the WHO 5 Moments poster. The objective of this study was to assess the use of emojis instead of the terms used in the WHO 5 moments poster and to understand whether they are appropriate to convey the hand hygiene indications. Accordingly, a new questionnaire was developed to evaluate the adequacy of the existing emojis to represent the main words used in the poster with emojis extracted from the Apple Color Emoji font set. To the best of our knowledge, no questionnaire has been developed before to assess the use of emojis in demonstrating IPC principles, and this is the first survey to evaluate the appropriateness of emojis in hand hygiene.

## Methods

Following a thorough review of the Unicode version 12.0 [17], the most applicable Apple emojis to the terms used in the WHO 5 Moments poster were extracted by the principal investigator (NL) (http://emojipedia.org/apple/). We developed a self-administered questionnaire to evaluate the proposed emojis for “hand”, “hygiene”, “touching”, “patient”, “clean”, “aseptic”, “procedure”, “body fluid”, “risk”, and “surroundings” (Additional file 1). We used a 4-point Likert scale ranging from 1 (to a great extent) to 4 (not at all) for each emoji to score the level of intuitiveness, scientific appropriateness, and social appropriateness of each of the proposed emojis. The following questions were used for each of the proposed emojis: “when you see the emoji, do you intuitively think of the proposed word?” to evaluate intuitiveness; “is the emoji a scientifically relevant substitute for the word it is replacing?” to assess scientific appropriateness; and “does the emoji have the quality of being socially proper to substitute the relevant word?” to evaluate social suitability.

Content validity was assessed to detect which emojis were relevant and could represent the specific word under assessment. Five experts including physicians, nurses, and a microbiologist who are working in the IPC team of the University of Geneva, primarily evaluated the proposed emojis for each word. The experts were asked to critically review the questionnaire and the proposed emojis, in order to select a list of emojis that may represent hand hygiene indications according to the WHO 5 Moments. They were also requested to provide written comments on the questionnaire components and design in order to improve the overall quality and representativeness of the items. We considered all the comments provided by the experts and modified the questionnaire as suggested. Eventually, 5 emojis were selected for each of the words; 43 different emojis were selected in total considering the repetition of some emojis as an option for other words or the use of two emojis to demonstrate one word. Most of the emojis belonged to the smileys and people category (21 out of 43) while 13 emojis were from the object’s category, 6 were from symbols category, and 3 were from nature emojis.

After scoring each emoji for each of the selected words, the respondents were asked to select their most and least favorite emoji in general. The questionnaire also included data regarding gender, age, level of education, specialty in IPC, country of origin, years active in IPC, frequency of using emojis in daily life (never, rarely, occasionally, at least one everyday, everyday in the majority of my messages that I send), type of used social media site (Facebook, YouTube, WhatsApp, Messenger, WeChat, Instagram, Twitter, Tumblr, LinkedIn, other(s) to be specified). The survey questionnaire is available in the Additional file 1.

Participants of the International Conference on Prevention and Infection Control (ICPIC), Geneva, held between 10 and 13 September, 2019, were invited via Email prior to the conference to complete the questionnaire. Hard copies of the questionnaires were also shared during the conference. All participants were informed that the information they provide would be kept confidential and individual answers would not be disclosed. A secure Email was created to collect questionnaire-based data for research. Accordingly, a total of 95 questionnaires were completed online or using hard copies of the printed questionnaire from May to October 2019. Completed questionnaires were collected and analysed to assess the suitability of the existing emojis to illustrate an emoji poster. Data from the questionnaire were saved in Excel sheets directly from the questionnaire forms. Descriptive statistics present frequencies, percentages along with 95% confidence intervals for the proportions or mean ± SD, as appropriate. All analyses were performed using R (version 3.6.3).

## Results

**Fig. 1:**
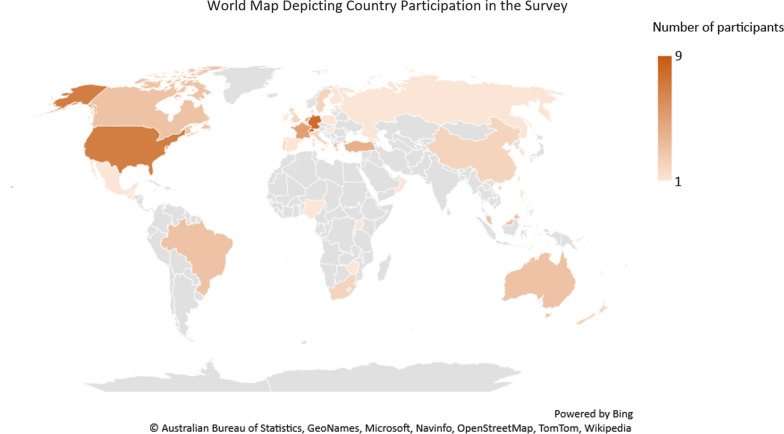
World map depicting country participation in the survey

**Fig. 2:**
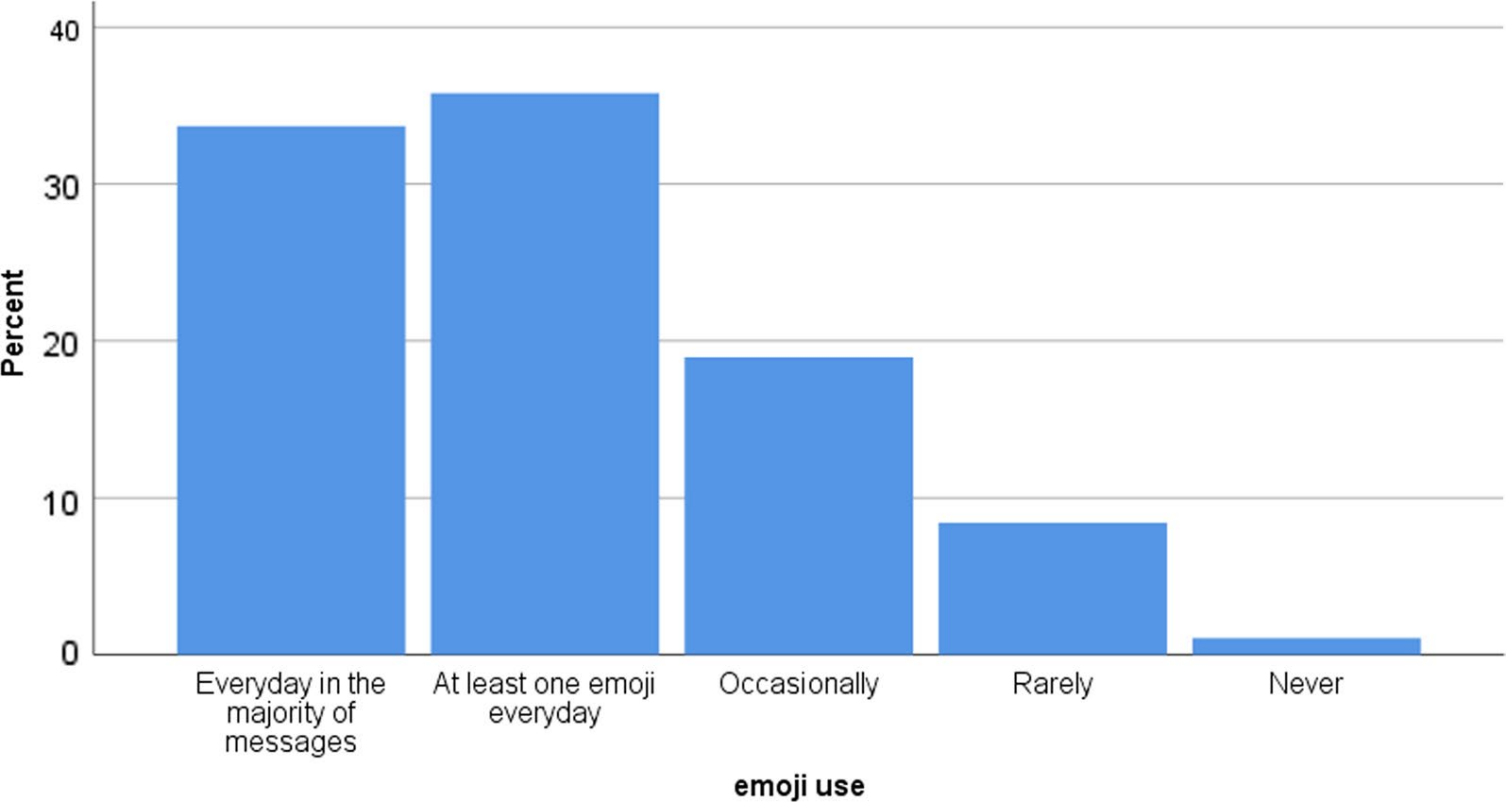
Frequency of using emojis among IPC practitioners participating in the survey

In total, 1264 participants attended ICPIC 2019 from different countries around the world [18], 95 of whom completed the survey. Of these, 63 (69%) of respondents were from countries with developed economies, 27 (29%) were from countries with developing economies, and 2 (2%) were from countries with economies in transition based on the UN country classification (Fig. 1). The majority of respondents (n = 69, 74%) were female. More than half 49 (54%) of respondents were physicians, 29 (32%) were nurses and 13 (14%) had other specialties in IPC. The mean age of the participants was 44.6 ± 10.87 years, with a mean active year in IPC of 12.3 ± 9.09. The frequency of using emojis among participants is shown in Fig. 2. The mean of the used social media platforms was 3.9 ± 1.88, among which WhatsApp was the most used platform (82.1%), followed by Facebook (61.0%) and LinkedIn (50.5%), while Tumblr had the lowest participation rate (1.0%).

Table 1 summarizes the assessment of the proposed emojis in terms of intuitiveness, scientific appropriateness and social appropriateness. Table 2 demonstrates the most and least preferred emojis for each of the words describing the WHO 5 Moments. Regarding the words “hand” and “hygiene”, most of the participants agreed on the use of  (44%) or  (32%) for hand, while none of the proposed hygiene-related emojis were appropriate according to them. For “Moment 1” and “Moment 4”, before and after touching a patient, we may be able to replace emojis with words as 72% of the participants agreed on the use of  for touching, and 63% agreed on  for patient. These moments may be shown as: “  hygiene  ” and “  hygiene”. As 53% of respondents agreed on  to show clean and 56% agreed on  to show procedure, “Moment 2”, known as “before clean/aseptic procedure” could also be shown as “  hygiene  ”. However there were no emojis to convey the word “aseptic”. Based on our findings, “Moment 3” which is described as “after body fluid exposure/risk” can also be demonstrated as: “  hygiene”, considering that 58% of participants agreed on  to show body fluids and 71% agreed on  to show risk. According to the participants, there are no emojis to show patient surroundings, hence it may not be possible to show “Moment 5” using emojis. The most appropriate emojis based on the total sum of scores were almost similar to the most favorite emojis except for “clean” and “surroundings” that were scored in favor of using  (mean ± SD: 5.98 ± 2.54), and  (mean ± SD: 6.05 ± 2.55), while chosen as  and  according to the participants’ most favorite emojis. Our findings showed limited associations between demographic data including gender, age, country and profession and overall sum of scores of the proposed emojis as well as the most and least favorite emojis.

**Table 1:**
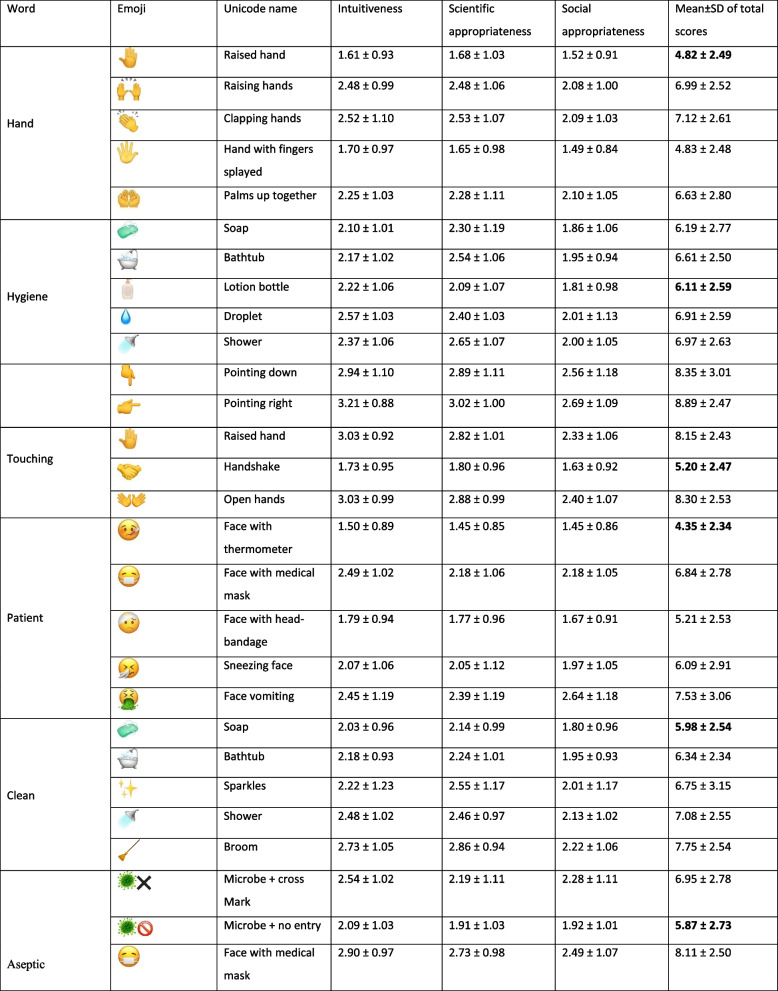

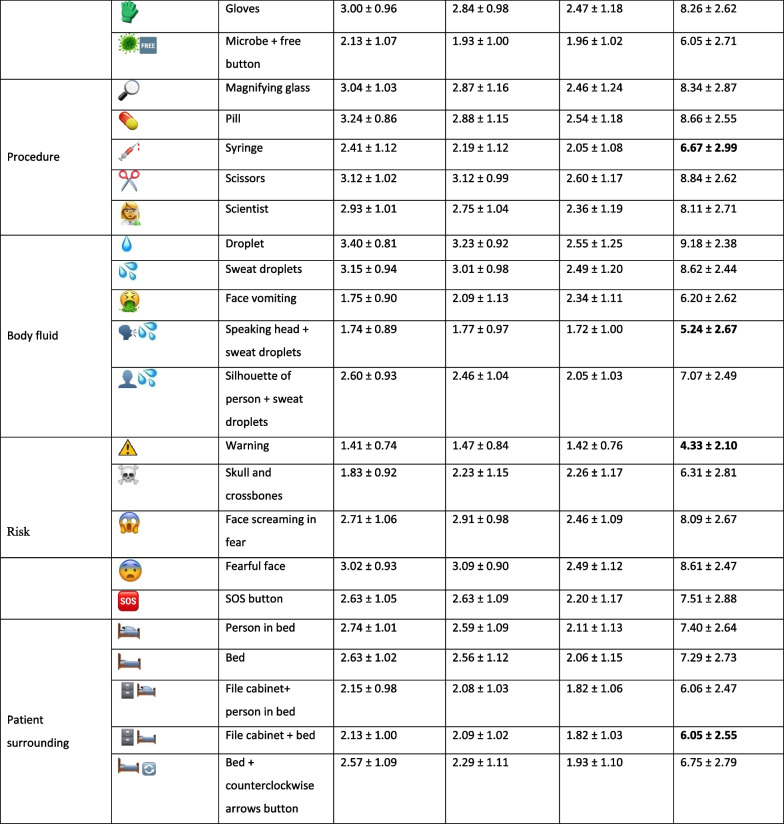
Evaluation of the scientific appropriateness, social appropriateness, and intuitiveness of the preselected emojis to express the WHO 5 Moments for hand hygiene action in healthcare, n = 95

Mean ± SD scores of scientific appropriateness, social appropriateness, and intuitiveness

**Table 2:**
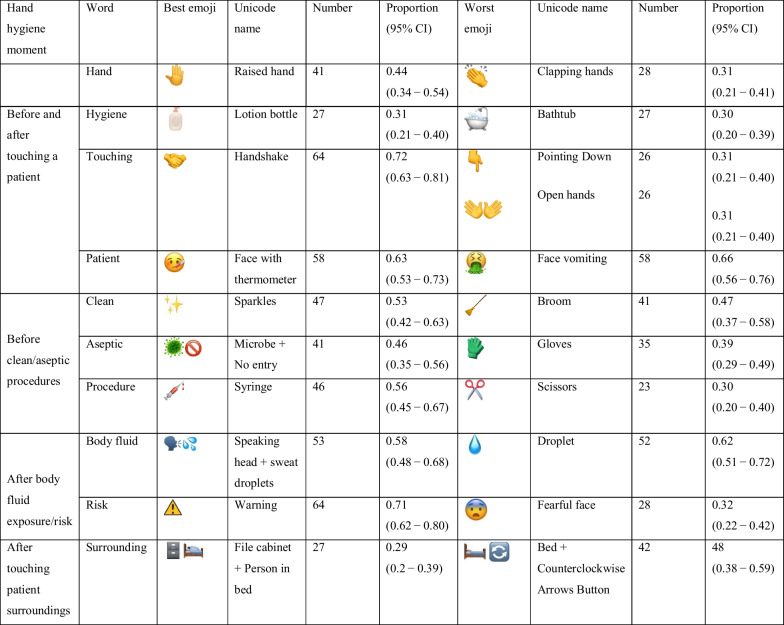
Hand hygiene in healthcare: the most and least favorite emojis to illustrate the WHO 5 Moments, n = 95

## Discussion

The emoji questionnaire gave a broad overview of IPC practitioners’ views regarding the capacity of emojis to replace the words used in the WHO 5 Moments poster. Considering the most favorite emojis selected for each word, 6 out of 10 words used in the WHO 5 Moments poster (including touching, patient, clean, procedure, body fluid, and risk) had a particular emoji by the majority of the respondents. The existing emojis proposed for the words “hand”, “hygiene”, “aseptic”, and “surrounding” seem to be less satisfactory. However, some of the emojis proposed for the words such as “hygiene” and “surrounding” had very similar mean scores which made it more difficult to decide which emoji was best suited to describe the word. For the word “hand”, as two of the proposed emojis were very similar in shape (  and  ), and were scored almost similarly, we could conclude that the existing emojis to show the word “hand” is appropriate.

Based on our results, women and men have the same understanding of the meaning of emojis, although it has been previously shown in the literature that women tend to use emojis more often compared to men [19]and there may be differences in interpretation of emojis between men and women. Similarly, we found no significant differences between gender and the most and least preferred emojis and also the overall sum of scores of each emoji. The use of emojis is also associated with age and younger people aged between 18 and 25 use emojis more frequently [19]. In our survey, differences related to age were limited and the participants agreed on most of the emojis independently from their age. Although cultural differences are known to affect emoji understanding [20, 21], we observed a few significant differences between countries and the overall sum of scores.

In another attempt to develop an emoji-based questionnaire, Marengo et al. tested a 10-item emoji-based instrument in order to assess depressive symptoms [22]. Based on the data collected from 1430 young adults, they found that 33 out of 36 emojis had significant correlations with the 10-item version of the Center for Epidemiologic Studies Depression Scale. They also concluded that this instrument was highly accurate in identifying depressed individuals. However, it is obvious that the existing emojis are mostly applicable in psychology, as there are various emojis to depict different emotions. When testing emojis in IPC, we realized that choosing emojis to demonstrate hand hygiene indications is much more complex.

Emojis have a number of advantages compared to other tools to help promote hand hygiene: they are used globally and are available for free on online social media platforms. They add emotion, attitude and attention when added to text, and there is a growing body of evidence that indicates that they can influence behaviour change [23]. Emojis enable people from different geographic regions that speak different languages to interact in a standardized manner using a set of compact single characters that bypass language barriers [24, 25]. To the best of our knowledge, this was the first survey to investigate the use of emojis to promote compliance with hand hygiene. According to our study, the existing emojis are insufficient to convey the WHO 5 Moments and better emojis are needed to depict some of the words used in the poster such as hand hygiene, aseptic, and surrounding environment. While hand hygiene with alcohol-based handrub is recognized as the cornerstone of IPC for preventing HAIs, there were no appropriate emojis to demonstrate this action which is the global standard of care [26]. Using the available emojis to depict “hand hygiene” might be confusing and not as straightforward as a dedicated emoji to this important IPC measure. During the COVID-19 pandemic, Twitter added a handwashing devoted emoji (  ) which seems more appropriate to depict this important action [27]. However, the limitation of this emoji is that it is mainly showing handwashing with water and soap, not necessarily hand hygiene with alcohol-based handrub. The limited use of emojis to demonstrate health-related concepts was also highlighted in the study of Adami and Cecchini, that prompted them to develop new emojis to depict the cardiopulmonary resuscitation steps [28].

The major limitation of our study was the low response rate in spite of the wide circulation of the questionnaire online and during an international conference, for five months. This could be due the required time to complete this questionnaire, as we asked different aspects of emoji appropriateness. We recommend the development of further questionnaires that are simpler and more user-friendly in order to assess the use of emojis in different topics. Most of the participants of this study were female and from developed countries, which could affect the evaluation of emojis. We may also need to increase awareness of both HCWs and the community regarding the impact of emojis as an integral part of social media on public health, as many participants were reluctant to complete the questionnaire as they believed using emojis is not acceptable in the medical literature.

One of the strengths of this study is that to the best of our knowledge, this is the first survey study to evaluate different emojis to represent for the words used in the WHO 5 Moments poster. This study provides insight regarding the possible role of emojis to remind HCWs to perform hand hygiene best practices. Given the different preferences for emojis across age groups, gender, and cultural backgrounds, future studies should investigate possible differences in the interpretation of emojis when used to demonstrate actions in healthcare.

## Conclusions

In summary, the findings of the current study indicate that the existing emojis may not be able to substitute for the words used in the WHO 5 Moments poster. Emojis might be a useful tool to address hand hygiene indications in healthcare that may eventually play a role in promoting this measure. However, emojis should be further studied in order to choose the most appropriate ones and avoid ambiguity and misinterpretation. We recommend further research in this area to evaluate the effect of using emojis in healthcare-related behaviours.

## Supplementary Information

**Additional file 1**. “Your Five Moments for Hand Hygiene” Emoji assessment.

## Data Availability

The datasets used and/or analysed during the current study are available from the corresponding author upon request.

## Abbreviations

HAI: Healthcare-associated infection
IPC: Infection prevention and control
WHO: World Health Organization
HCWs: Healthcare workers
