# Supplementary material for: Are emojis ready to promote the WHO 5 moments for hand hygiene in healthcare?
Source: Antimicrob Resist Infect Control. 2022 Oct 26;11:127. doi: 10.1186/s13756-022-01164-1 (PMC9607812; doi:10.1186/s13756-022-01164-1)
Supplement: Supplementary file 1 — Additional file 1. “Your Five Moments for Hand Hygiene” Emoji assessment. [file 13756_2022_1164_MOESM1_ESM.pdf]

Hôpitaux Universitaires Genève
University of Geneva Hospitals and Faculty of Medicine, Geneva, Switzerland

UNIVERSITÉ DE GENÈVE
FACULTÉ DE MÉDECINE

WHO Collaborating Centre on Patient Safety Infection Control & Improving Practices

Name: ……………………………… Date: ………………………

"Your Five Moments for Hand Hygiene" Emoji Assessment

Prepared by: Dr. Nasim Lotfinejad

Under supervision of: Prof. Didier Pittet

Emojis have become popular worldwide as online interaction becomes increasingly prevalent in daily life. Studies have proven that users adapt as technology improves, and emojis are an example of this adaptation as a replacement for the nonverbal cues which play a major role in effective communication. Keeping in mind that emotional events are remembered more accurately and for a longer duration than neutral events, generating a visual description is an effective method to optimize information retention.

This questionnaire intends to collect data from Infection Prevention and Control (IPC) experts regarding the appropriateness of existing emojis for depicting the WHO "Five Moments for Hand Hygiene". Accordingly, among currently existing emojis a set has been suggested for almost every word used in the Five Moments including hand, hygiene, touching, patient, clean, aseptic, procedure, body fluid, risk, and surroundings. Please indicate the level of intuitiveness, scientific appropriateness, and social appropriateness of the emojis using a scale from **1 (to a great extent) to 4 (not at all)** for each emoji. All information will be kept confidential and your individual answers will not be disclosed. Should you have any comments or questions, please feel free to contact Dr. Nasim Lotfinejad at Nasim.lotfinezhad@gmail.com.

Thank you very much for your time and cooperation.

Demographics:

Gender: Male ☐ Female ☐

Age in years: ………….

Level of education: ……………………….

Specialty in IPC: Doctor ☐ Nurse ☐ PhD ☐ Other: ……………..

Years active in IPC: ……………………….

Country of origin: ………………………….

How often do you use emojis in your daily life?

Never ☐ Rarely ☐ Occasionally ☐ At least one every day ☐

Every day in the majority of my messages that I send ☐

Which social media sites are you using? (Please indicate all you have used)

Facebook ☐ YouTube ☐ WhatsApp ☐ Messenger ☐ WeChat ☐

Instagram ☐ Twitter ☐ Tumblr ☐ LinkedIn ☐ Other: …………………

Please rate the following emojis in terms of:

1) **Intuitiveness**: when you see the emoji, do you intuitively think of the proposed word?
2) **Scientific appropriateness**: is the emoji a scientifically relevant substitute for the word it is replacing?
3) **Social appropriateness**: does the emoji have the quality of being socially proper to substitute the relevant word?

1) "Hand": 1= to a great extent, 2= somewhat, 3= very little, 4= not at all

	Is this emoji intuitive?				Is this emoji socially appropriate?				Is this emoji scientifically appropriate?			
**Hand**	**1**	**2**	**3**	**4**	**1**	**2**	**3**	**4**	**1**	**2**	**3**	**4**
1) ✋	☐	☐	☐	☐	☐	☐	☐	☐	☐	☐	☐	☐
2) 🙌	☐	☐	☐	☐	☐	☐	☐	☐	☐	☐	☐	☐
3) 👏	☐	☐	☐	☐	☐	☐	☐	☐	☐	☐	☐	☐
4) 🖐	☐	☐	☐	☐	☐	☐	☐	☐	☐	☐	☐	☐
5) 🤲	☐	☐	☐	☐	☐	☐	☐	☐	☐	☐	☐	☐

Which is your most favorite "Hand" emoji?

✋ ☐ 🙌 ☐ 👏 ☐ 🖐 ☐ 🤲 ☐

Which is your least favorite "Hand" emoji?

✋ ☐ 🙌 ☐ 👏 ☐ 🖐 ☐ 🤲 ☐

Is the most preferred emoji appropriate? **Yes** ☐ **No** ☐

If No, please explain here:

……………………………………………….

Are there any comments or suggestions of what a better emoji would look like?

2) “Hygiene”: 1= to a great extent, 2= somewhat, 3= very little, 4= not at all

	Is this emoji intuitive?				Is this emoji socially appropriate?				Is this emoji scientifically appropriate?			
**Hygiene**	**1**	**2**	**3**	**4**	**1**	**2**	**3**	**4**	**1**	**2**	**3**	**4**
1) 🧼	☐	☐	☐	☐	☐	☐	☐	☐	☐	☐	☐	☐
2) 🛁	☐	☐	☐	☐	☐	☐	☐	☐	☐	☐	☐	☐
3) 🧴	☐	☐	☐	☐	☐	☐	☐	☐	☐	☐	☐	☐
4) 💧	☐	☐	☐	☐	☐	☐	☐	☐	☐	☐	☐	☐
5) 🚿	☐	☐	☐	☐	☐	☐	☐	☐	☐	☐	☐	☐

Which is your most favorite “Hygiene” emoji?

🧼 ☐ 🛁 ☐ 🧴 ☐ 💧 ☐ 🚿 ☐

Which is your least favorite “Hygiene” emoji?

🧼 ☐ 🛁 ☐ 🧴 ☐ 💧 ☐ 🚿 ☐

Is the most preferred emoji appropriate? **Yes** ☐ **No** ☐

If No, please explain here:

……………………………………………….

Are there any comments or suggestions of what a better emoji would look like?

3) “Touching”: 1= to a great extent, 2= somewhat, 3= very little, 4= not at all

	Is this emoji intuitive?				Is this emoji socially appropriate?				Is this emoji scientifically appropriate?			
**Touching**	1	2	3	4	1	2	3	4	1	2	3	4
1) 👇	☐	☐	☐	☐	☐	☐	☐	☐	☐	☐	☐	☐
2) 👉	☐	☐	☐	☐	☐	☐	☐	☐	☐	☐	☐	☐
3) ✋	☐	☐	☐	☐	☐	☐	☐	☐	☐	☐	☐	☐
4) 🤝	☐	☐	☐	☐	☐	☐	☐	☐	☐	☐	☐	☐
5) 👐	☐	☐	☐	☐	☐	☐	☐	☐	☐	☐	☐	☐

Which is your most favorite “Touching” emoji?

👇 ☐ 👉 ☐ ✋ ☐ 🤝 ☐ 👐 ☐

Which is your least favorite “Touching” emoji?

👇 ☐ 👉 ☐ ✋ ☐ 🤝 ☐ 👐 ☐

Is the most preferred emoji appropriate? **Yes** ☐ **No** ☐

If No, please explain here:

……………………………………………………

Are there any comments or suggestions of what a better emoji would look like?

4) “Patient”: 1= to a great extent, 2= somewhat, 3= very little, 4= not at all

	Is this emoji intuitive?				Is this emoji socially appropriate?				Is this emoji scientifically appropriate?			
**Patient**	1	2	3	4	1	2	3	4	1	2	3	4
1) 🤒	☐	☐	☐	☐	☐	☐	☐	☐	☐	☐	☐	☐
2) 😷	☐	☐	☐	☐	☐	☐	☐	☐	☐	☐	☐	☐
3) 🤕	☐	☐	☐	☐	☐	☐	☐	☐	☐	☐	☐	☐
4) 🤧	☐	☐	☐	☐	☐	☐	☐	☐	☐	☐	☐	☐
5) 🤮	☐	☐	☐	☐	☐	☐	☐	☐	☐	☐	☐	☐

Which is your most favorite “Patient” emoji?

🤒 ☐ 😷 ☐ 🤕 ☐ 🤧 ☐ 🤮 ☐

Which is your least favorite “Patient” emoji?

🤒 ☐ 😷 ☐ 🤕 ☐ 🤧 ☐ 🤮 ☐

Is the most preferred emoji appropriate? **Yes** ☐ **No** ☐

If No, please explain here:

……………………………………………

Are there any comments or suggestions of what a better emoji would look like?

5) “Clean”: 1= to a great extent, 2= somewhat, 3= very little, 4= not at all

	Is this emoji intuitive?			
**Clean**	**1**	**2**	**3**	**4**
1) 🧼	☐	☐	☐	☐
2) 🛁	☐	☐	☐	☐
3) ✨	☐	☐	☐	☐
4) 🚿	☐	☐	☐	☐
5) 🧹	☐	☐	☐	☐

Is this emoji socially appropriate?			
**1**	**2**	**3**	**4**
☐	☐	☐	☐
☐	☐	☐	☐
☐	☐	☐	☐
☐	☐	☐	☐
☐	☐	☐	☐

Is this emoji scientifically appropriate?			
**1**	**2**	**3**	**4**
☐	☐	☐	☐
☐	☐	☐	☐
☐	☐	☐	☐
☐	☐	☐	☐
☐	☐	☐	☐

Which is your most favorite “Clean” emoji?

🧼 ☐ 🛁 ☐ ✨ ☐ 🚿 ☐ 🧹 ☐

Which is your least favorite “Clean” emoji?

🧼 ☐ 🛁 ☐ ✨ ☐ 🚿 ☐ 🧹 ☐

Is the most preferred emoji appropriate? **Yes** ☐ **No** ☐

If No, please explain here:

………………………………………………

Are there any comments or suggestions of what a better emoji would look like?

6) “Aseptic”: 1= to a great extent, 2= somewhat, 3= very little, 4= not at all

	Is this emoji intuitive?				Is this emoji socially appropriate?				Is this emoji scientifically appropriate?			
**Aseptic**	**1**	**2**	**3**	**4**	**1**	**2**	**3**	**4**	**1**	**2**	**3**	**4**
1) 🦠❌	☐	☐	☐	☐	☐	☐	☐	☐	☐	☐	☐	☐
2) 🦠🚫	☐	☐	☐	☐	☐	☐	☐	☐	☐	☐	☐	☐
3) 😷	☐	☐	☐	☐	☐	☐	☐	☐	☐	☐	☐	☐
4) 🧤	☐	☐	☐	☐	☐	☐	☐	☐	☐	☐	☐	☐
5) 🦠🆓	☐	☐	☐	☐	☐	☐	☐	☐	☐	☐	☐	☐

Which is your most favorite “Aseptic” emoji?

🦠❌ ☐ 🦠🚫 ☐ 😷 ☐ 🧤 ☐ 🦠🆓 ☐

Which is your least favorite “Aseptic” emoji?

🦠❌ ☐ 🦠🚫 ☐ 😷 ☐ 🧤 ☐ 🦠🆓 ☐

Is the most preferred emoji appropriate? **Yes** ☐ **No** ☐

If No, please explain here:

……………………………………………….

Are there any comments or suggestions of what a better emoji would look like?

7) “Procedure”: 1= to a great extent, 2= somewhat, 3= very little, 4= not at all

	Is this emoji intuitive?				Is this emoji socially appropriate?				Is this emoji scientifically appropriate?			
**Procedure**	1	2	3	4	1	2	3	4	1	2	3	4
1) 🔍	☐	☐	☐	☐	☐	☐	☐	☐	☐	☐	☐	☐
2) 💊	☐	☐	☐	☐	☐	☐	☐	☐	☐	☐	☐	☐
3) 💉	☐	☐	☐	☐	☐	☐	☐	☐	☐	☐	☐	☐
4) ✂️	☐	☐	☐	☐	☐	☐	☐	☐	☐	☐	☐	☐
5) 👩‍🔬	☐	☐	☐	☐	☐	☐	☐	☐	☐	☐	☐	☐

Which is your most favorite “Procedure” emoji?

🔍 ☐ 💊 ☐ 💉 ☐ ✂️ ☐ 👩‍🔬 ☐

Which is your least favorite “Procedure” emoji?

🔍 ☐ 💊 ☐ 💉 ☐ ✂️ ☐ 👩‍🔬 ☐

Is the most preferred emoji appropriate? Yes ☐ No ☐

If No, please explain here:

………………………………………………

Are there any comments or suggestions of what a better emoji would look like?

8) “Body fluid”: 1= to a great extent, 2= somewhat, 3= very little, 4= not at all

	Is this emoji intuitive?				Is this emoji socially appropriate?				Is this emoji scientifically appropriate?			
**Body fluid**	**1**	**2**	**3**	**4**	**1**	**2**	**3**	**4**	**1**	**2**	**3**	**4**
1) 💧	☐	☐	☐	☐	☐	☐	☐	☐	☐	☐	☐	☐
2) 💦	☐	☐	☐	☐	☐	☐	☐	☐	☐	☐	☐	☐
3) 🤮	☐	☐	☐	☐	☐	☐	☐	☐	☐	☐	☐	☐
4) 🗣💦	☐	☐	☐	☐	☐	☐	☐	☐	☐	☐	☐	☐
5) 👤💦	☐	☐	☐	☐	☐	☐	☐	☐	☐	☐	☐	☐

Which is your most favorite “Body fluid” emoji?

💧 ☐ 💦 ☐ 🤮 ☐ 🗣💦 ☐ 👤💦 ☐

Which is your least favorite “Body fluid” emoji?

💧 ☐ 💦 ☐ 🤮 ☐ 🗣💦 ☐ 👤💦 ☐

Is the most preferred emoji appropriate? **Yes** ☐ **No** ☐

If No, please explain here:

……………………………………………….

Are there any comments or suggestions of what a better emoji would look like?

9) “Risk”: 1= to a great extent, 2= somewhat, 3= very little, 4= not at all

	Is this emoji intuitive?				Is this emoji socially appropriate?				Is this emoji scientifically appropriate?			
**Risk**	**1**	**2**	**3**	**4**	**1**	**2**	**3**	**4**	**1**	**2**	**3**	**4**
1) ⚠️	☐	☐	☐	☐	☐	☐	☐	☐	☐	☐	☐	☐
2) ☠️	☐	☐	☐	☐	☐	☐	☐	☐	☐	☐	☐	☐
3) 😱	☐	☐	☐	☐	☐	☐	☐	☐	☐	☐	☐	☐
4) 😨	☐	☐	☐	☐	☐	☐	☐	☐	☐	☐	☐	☐
5) 🆘	☐	☐	☐	☐	☐	☐	☐	☐	☐	☐	☐	☐

Which is your most favorite “Risk” emoji?

⚠️ ☐ ☠️ ☐ 😱 ☐ 😨 ☐ 🆘 ☐

Which is your least favorite “Risk” emoji?

⚠️ ☐ ☠️ ☐ 😱 ☐ 😨 ☐ 🆘 ☐

Is the most preferred emoji appropriate? **Yes** ☐ **No** ☐

If No, please explain here:

……………………………………………………

Are there any comments or suggestions of what a better emoji would look like?

10) **“Surroundings”: 1= to a great extent, 2= somewhat, 3= very little, 4= not at all**

	Is this emoji intuitive?				Is this emoji socially appropriate?				Is this emoji scientifically appropriate?			
**Surroundings**	**1**	**2**	**3**	**4**	**1**	**2**	**3**	**4**	**1**	**2**	**3**	**4**
1) 🛌	☐	☐	☐	☐	☐	☐	☐	☐	☐	☐	☐	☐
2) 🛏	☐	☐	☐	☐	☐	☐	☐	☐	☐	☐	☐	☐
3) 🗄🛌	☐	☐	☐	☐	☐	☐	☐	☐	☐	☐	☐	☐
4) 🗄🛏	☐	☐	☐	☐	☐	☐	☐	☐	☐	☐	☐	☐
5) 🛏🔄	☐	☐	☐	☐	☐	☐	☐	☐	☐	☐	☐	☐

Which is your most favorite “Surroundings” emoji?

🛌 ☐ 🛏 ☐ 🗄🛌 ☐ 🗄🛏 ☐ 🛏🔄 ☐

Which is your least favorite “Surroundings” emoji?

🛌 ☐ 🛏 ☐ 🗄🛌 ☐ 🗄🛏 ☐ 🛏🔄 ☐

Is the most preferred emoji appropriate? **Yes** ☐ **No** ☐

If No, please explain here:

…………………………………………….

Are there any comments or suggestions of what a better emoji would look like?
